# Sertraline-Induced Acute Eosinophilic Pneumonia

**DOI:** 10.7759/cureus.12022

**Published:** 2020-12-11

**Authors:** Prakash Adhikari, Krystal Alexander, Ademayowa O Ademiluyi, James Appiah-Pippim

**Affiliations:** 1 Internal Medicine, Piedmont Athens Regional Medical Center, Athens, USA; 2 Pulmonary and Critical Care Medicine, Piedmont Athens Regional Medical Center, Athens, USA

**Keywords:** acute eosinophilic pneumonia, ssri, sertraline, eosinophilic pneumonia, antidepressants

## Abstract

Acute eosinophilic pneumonia (AEP) is a rare but severe respiratory syndrome characterized by fever, hypoxemic respiratory failure, diffuse pulmonary infiltrates, and pulmonary eosinophilia. The most common cause of AEP is idiopathic, but it can be associated with antidepressant medications like sertraline.

A 76-year-old female presented to our ED with acute hypoxemic respiratory failure. She had no history of smoking or prior lung disease. She did not improve after treatment with broad spectrum antibiotics so a trial of corticosteroids was initiated. Her work-up was negative for infectious or collagen vascular causes of the respiratory failure. She was diagnosed with AEP associated with sertraline. Her condition improved with corticosteroid therapy after discontinuation of sertraline. This case report highlights AEP as a possible adverse reaction of sertraline. Prompt discontinuation of the offending drug is necessary for early recovery.

## Introduction

Eosinophilic lung diseases are rare lung conditions which are characterized by accumulation of eosinophils in the alveolar space and the lung parenchyma. Eosinophilic granulomatosis with polyangiitis and hyper-eosinophilic syndrome are eosinophilic lung disease with systemic involvement whereas acute eosinophilic pneumonia (AEP), chronic eosinophilic pneumonia, and Loeffler syndrome tend to involve only the lung. Eosinophilic lung diseases can be primary or secondary depending on the absence or presence of a known causative factor [1].

Acute eosinophilic pneumonia was first described in 1989 by Allen and Davis who reported a case series of an idiopathic lung disease characterized by febrile illness, diffuse pulmonary infiltrate, and pulmonary eosinophilia [2]. The epidemiology of this condition remains understudied because of its rarity. It typically occurs in previously healthy young adults and predominantly in males. There are no studies showing relation with other allergic condition like asthma, atopic dermatitis, or allergic rhinitis. However, it is common in smokers [3]. Common clinical symptoms include dyspnea, cough, fever, chills, and chest pain which can result in rapidly progressive respiratory failure, sometimes requiring mechanical ventilation. CT scan of lung typically shows bilateral ground glass opacities. These opacities are usually present in the upper lung zones and they tend to migrate in 25% of patients [4]. There are no consensus guidelines for the diagnosis of AEP. Diagnosis is made by supportive symptoms and signs along with eosinophilia (≥25%) in bronchoalveolar lavage or infiltration of eosinophils in lung parenchyma seen on lung biopsy. The duration of illness from the onset of symptoms is typically less than a month. Most cases of AEP are idiopathic. Identification of causative factors is very important for successful treatment. Avoidance of causative factors along with corticosteroid is very effective in treatment and usually results in complete recovery.

Several case reports have identified a number of drugs and environmental factors as possible causes of secondary AEP. A metanalysis by Bartal et al. documented 228 cases of drug-induced AEP reported between 1990 and 2017. There were 32 cases each of mesalamine- and daptomycin-induced AEP. They also reported three cases of venlafaxine-induced and one case each of paroxetine-, duloxetine-, and sertraline-induced AEP [5]. In this case report we present a case of sertraline-induced AEP.

## Case presentation

**Figure 1:**
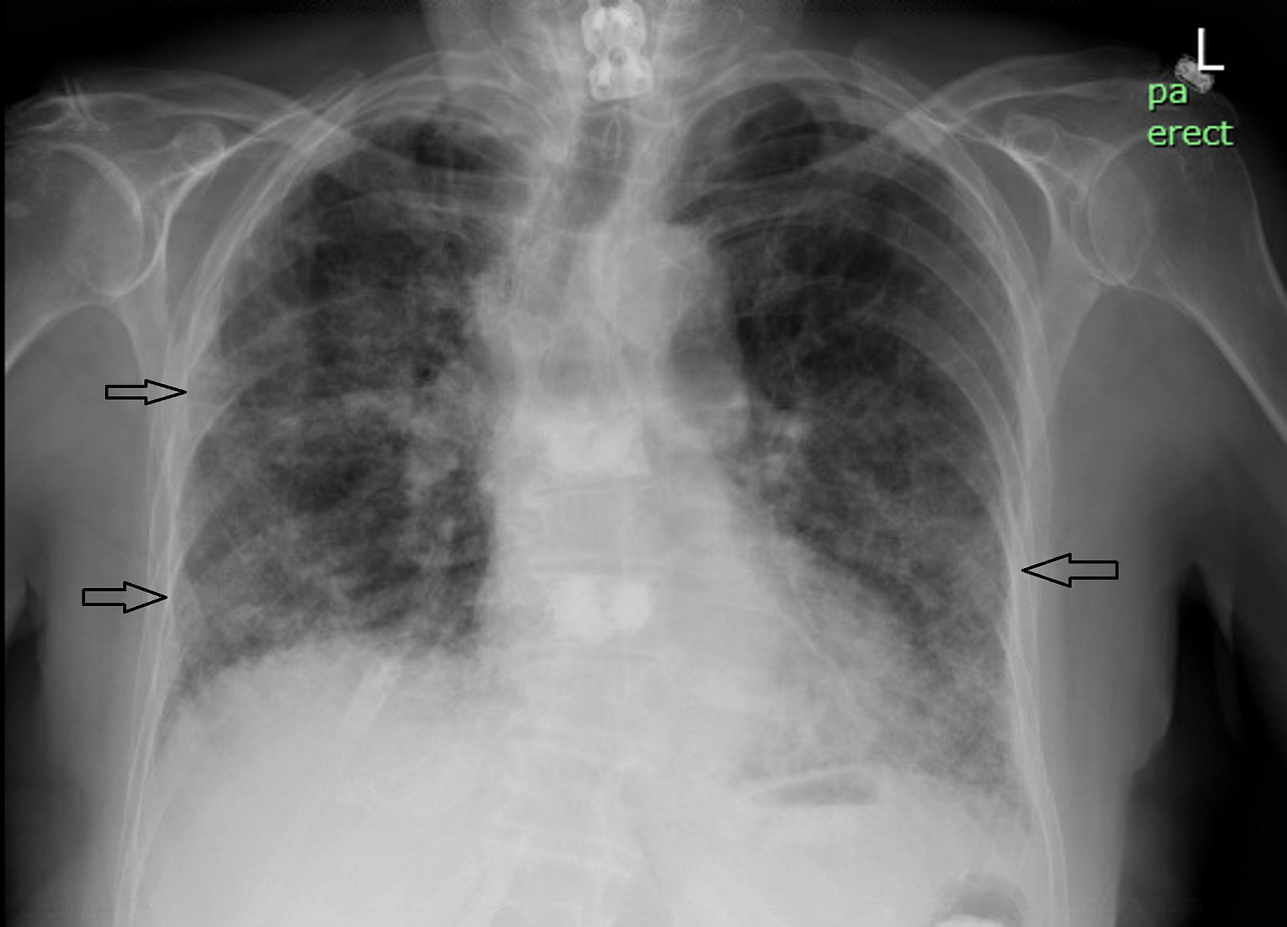
Chest X-ray. Arrows showing diffuse interstitial opacity

**Figure 2:**
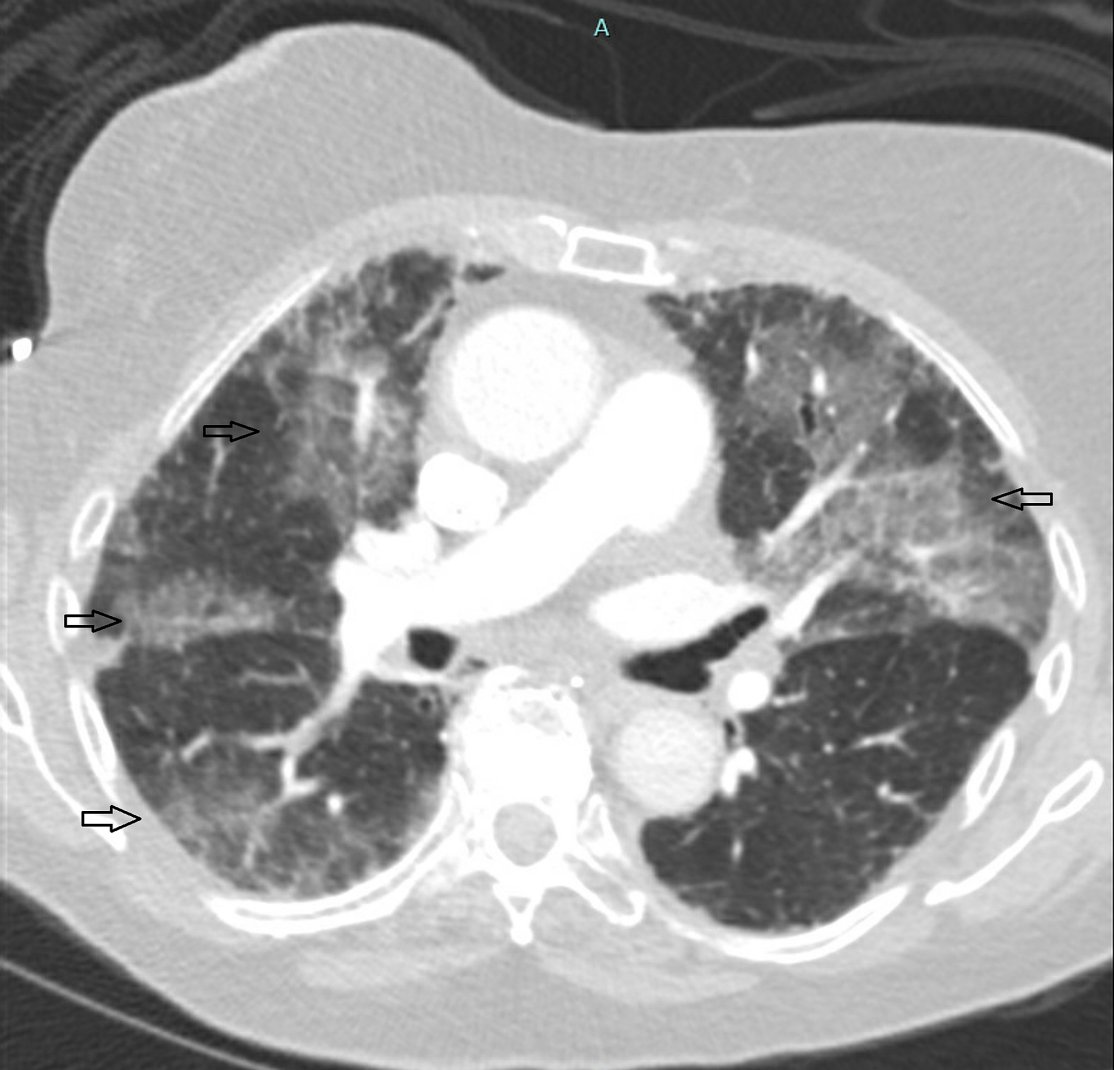
Contrast CT scan chest. Arrows showing patchy areas of ground glass opacity

A 76-year-old female with history of hypertension, hyperlipidemia, and depression presented with complaints of progressive dyspnea, fever, and dry cough for a week. She had no history of smoking, recent ill contacts or travel. Initial labs showed normal complete blood count (CBC), normal complete metabolic panel (CMP), and elevated C reactive protein (>8). Chest X-ray showed diffuse interstitial opacity (Figure 1). Tests for COVID-19, mycoplasma, legionella, and streptococcus were negative. CT angiography chest ruled out pulmonary embolism and showed patchy areas of ground glass opacity in bilateral lung field (Figure 2). As her condition worsened, she required high flow oxygen and was transferred to the ICU. Empiric antibiotic coverage initially with ceftriaxone and azithromycin did not improve her symptoms. Antibiotics were switched to piperacillin-tazobactam and a stress dose methylprednisolone 60 mg daily was also added. Acid fast bacilli (AFB), viral and fungal cultures were all negative. Tests for connective tissue disease were also negative. Bronchoalveolar lavage done revealed eosinophils of 25%. A diagnosis of AEP was made.

Antibiotics were discontinued. Pulse therapy with methylprednisolone 1 g per day was begun but she did not improve after three days. A careful review of her medications showed that she had been on sertraline 50 mg daily which was increased to 200 mg daily two months prior. Sertraline was discontinued because it is associated with AEP. Prednisolone 60 mg daily was continued. The patient's condition subsequently improved, and she was discharged on fourth day after discontinuation of sertraline with prednisolone 60 mg.

## Discussion

Diagnosis of AEP is made with acute onset of symptoms of less than seven days before presentation, fever, bilateral infiltrates on chest radiograph, severe hypoxemia (PaO2 on room air < 60, O2 saturation on room air <90%) and eosinophilia in bronchoalveolar lavage fluid showing ≥25% eosinophils [1]. These patients may not always have peripheral eosinophilia. Lung biopsy is often not necessary for diagnosis but maybe helpful if the diagnosis remains unclear. Most bacterial pneumonia can be ruled out by the presence of eosinophilia; however, *Pneumocystis jiroveci* pneumonia, fungal pneumonia, and parasitic infections such as *Strongyloides stercoralis*, and filariasis can present with eosinophilia [1].

The exact etiology of acute eosinophilia is unknown. It has been hypothesized that it involves an acute hypersensitivity reaction to inhaled antigens such as tobacco smoke. It can also develop under unusual outdoor circumstances like military personnel working in the Middle Eastern Desert [6], in firefighters following the collapse of the World Trade Center towers [7], and inhalation of smoke from fireworks [8]. Many medications have been implicated in drug-induced AEP. The commonly associated drugs are daptomycin, mesalamine, sulfasalazine, and minocycline [5]. There are very few case reports documenting antidepressant-induced AEP. One such report was that of AEP in context of clomipramine and sertraline use [9]. Another report linked venlafaxine use to AEP in a patient who was previously on sertraline [10]. Two other case reports documented AEP secondary to sertraline [11]. In all of these cases discontinuation of sertraline and other associated medications along with corticosteroid use resulted in resolution of the symptoms.

Very little has been understood in terms of pathophysiology of AEP in the setting of selective serotonin reuptake inhibitor (SSRI). Research has demonstrated that serotonin acts as an immune modulator and modulates cytokines secretion, neutrophil recruitment, and T-cell activation. Serotonin also acts as a chemotactic factor for eosinophils through 5-hydroxy-tryptamine 2A (5HT2A) receptor. It mediates eosinophil recruitment and results in airway inflammation and hyper-responsiveness [12-13]. One study also demonstrated that serotonin has a role in smooth muscle contraction [14]. Our patient was on multiple medications. Sertraline was the only medication that had been implicated before in AEP. The recent increase in the dose of sertraline, and lack of improvement of symptoms with pulse steroid therapy for three days prior to discontinuation of sertraline and rapid recovery after discontinuation of sertraline highly suggests the diagnosis of sertraline-induced AEP.

## Conclusions

Acute eosinophilic pneumonia is a rare condition often confused with infectious pneumonia. Failure to improve clinically with standard therapy should prompt physicians to think if AEP is a possible cause of respiratory failure. There are several medications associated with AEP including SSRI. Careful review of medications can help identify potential culprit medications. Early discontinuation of the potential offending medication along with steroid therapy is the treatment of choice for AEP.
